# Interleukin-17 Is Required for Control of Chronic Lung Infection Caused by Pseudomonas aeruginosa

**DOI:** 10.1128/IAI.00717-16

**Published:** 2016-11-18

**Authors:** Hannah K. Bayes, Neil D. Ritchie, Thomas J. Evans

**Affiliations:** Institute of Infection, Immunity and Inflammation, University of Glasgow, Glasgow, United Kingdom; The University of Massachusetts Medical School

## Abstract

Chronic pulmonary infection with Pseudomonas aeruginosa is a feature of cystic fibrosis (CF) and other chronic lung diseases. Cytokines of the interleukin-17 (IL-17) family have been proposed as important in the host response to P. aeruginosa infection through their role in augmenting antibacterial immune responses, although their proinflammatory effect may contribute to lung damage that occurs as a result of chronic infection. We set out to explore the role of IL-17 in the host response to chronic P. aeruginosa infection. We used a murine model of chronic pulmonary infection with CF-related strains of P. aeruginosa. We demonstrate that IL-17 cytokine signaling is essential for mouse survival and prevention of chronic infection at 2 weeks postinoculation using two different P. aeruginosa strains. Following infection, there was a marked expansion of cells within mediastinal lymph nodes, comprised mainly of innate lymphoid cells (ILCs); ∼90% of IL-17-producing (IL-17^+^) cells had markers consistent with group 3 ILCs. A smaller percentage of IL-17^+^ cells had markers consistent with a B1 phenotype. In lung homogenates harvested 14 days following infection, there was a significant expansion of IL-17^+^ cells; about 50% of these were CD3^+^, split equally between CD4^+^ Th17 cells and γδ T cells, while the CD3^−^ IL-17^+^ cells were almost exclusively group 3 ILCs. Further experiments with B cell-deficient mice showed that B cell production of IL-17 or natural antibodies did not provide any defense against chronic P. aeruginosa infection. Thus, IL-17 rather than antibody is a key element in host defense against chronic pulmonary infection with P. aeruginosa.

## INTRODUCTION

Some bacteria have evolved the ability to produce chronic infection of the respiratory tract. Mycobacterium tuberculosis is perhaps the best known example, but the Gram-negative pathogen Pseudomonas aeruginosa can also become persistent in the lower airways. This occurs most notably in patients with cystic fibrosis (CF) and bronchiectasis but is also increasingly recognized in other chronic lung diseases, such as chronic obstructive pulmonary disease (COPD). In CF, P. aeruginosa infections are initially intermittent and can be eradicated by intensive antibiotic treatment (1). Transition to chronic P. aeruginosa airway infection usually ensues, such that by age 20, 60 to 70% of CF patients are chronically infected (2). The continuous presence of P. aeruginosa in the airways is accompanied by an inexorable decline in respiratory function, leading to premature death or lung transplantation (3). Thus, this switch from intermittent to chronic infection is a key event in the progression of disease (1). Although antibiotics can delay this transition, better therapies aimed at preventing chronic infection could potentially significantly attenuate the rate of decline in lung function in patients affected by CF, as well in other chronic lung diseases in which chronic P. aeruginosa infection occurs.

Little is known of the mechanisms of host defense against chronic P. aeruginosa infection. Cytokines of the interleukin-17 (IL-17) family have been suggested as important in protection against P. aeruginosa infection. IL-17 in the lung may be important in host defense against P. aeruginosa through its ability to orchestrate a neutrophil response and by the induction of a variety of innate antimicrobial peptides (4). Increased levels of IL-17A (hereinafter referred to as IL-17) are found in sputum and bronchial lavage specimens of patients with CF (5), produced by a variety of cells of the innate and acquired immune system, including T cells of the Th17 lineage (6–9). Other cells known to produce IL-17 include, *inter alia*, innate lymphoid cells (ILCs), γδ T cells, and natural killer (NK) cells. Although such inflammatory responses can contribute to host defense, they also can potentially cause tissue damage, as is well known for M. tuberculosis infection, where the host inflammatory response can result in significant tissue damage. Proinflammatory actions of IL-17 in P. aeruginosa infection could increase tissue damage through excess neutrophil accumulation and induction of matrix metalloproteinases (10). Indeed, the inflammatory changes and subsequent bronchiectasis so typical of CF have been suggested to be driven by IL-17 cytokines. Although one study examined the role of IL-17 in acute infection (11), the specific role of IL-17 in chronic P. aeruginosa infection has not been addressed.

In the work presented here, we have defined the interactions and effector functions of the IL-17 axis in the pathogenesis of chronic pulmonary P. aeruginosa infection. Using a murine model, we show that IL-17 signaling is crucial in host defense against chronic P. aeruginosa infection, protecting against chronic colonization and death. Despite increased bacterial burdens, mice lacking IL-17 signaling had less weight loss than controls. We identified a diverse range of cellular sources of IL-17 both in draining mediastinal lymph nodes and in lungs following infection.

## MATERIALS AND METHODS

### Agar bead infection model.

The infection model was adapted from the protocol described by van Heeckeren and Schluchter (12) and modified as described previously (13). Pseudomonas aeruginosa-laden agar beads were prepared the day before inoculation and stored overnight at 4°C, and a different bead preparation was used for each experiment. P. aeruginosa-laden beads were stored on ice throughout the murine surgery. Following inoculation of P. aeruginosa-laden beads, the inoculum administered was confirmed by homogenization and quantitative bacteriology of a further two aliquots of beads. Sterile agar beads were stored at 4°C and used for several experiments. Sterile agar bead preparations were confirmed to be sterile before and after each use. For experiments using knockout mutants, all animals (knockout mutants and wild-type [WT] controls) were treated with P. aeruginosa-laden beads. In separate experiments, WT mice were treated with either P. aeruginosa-laden or sterile agar beads.

For inoculation with P. aeruginosa-laden beads, mice were anesthetized using isoflurane via nose cone, and the trachea was exposed and cannulated (22-gauge intravenous cannulae; BD Biosciences) under aseptic conditions. An average inoculum of 1 × 10^6^ CFU/50 μl per mouse was delivered. Animals were closely monitored postoperatively using a disease severity scoring system (see Table S1 in the supplemental material). An animal reaching a moribund endpoint was euthanized.

Surviving animals at 14 days after infection were culled; lungs were removed from some and mechanically homogenized, and aliquots plated on bacteriological medium to determine the numbers of CFU of P. aeruginosa. Chronicity rates were defined as the percentages of animals at 14 days after infection that had viable P. aeruginosa cells recovered from their lungs using this method.

### Flow cytometry.

Antibodies to the following were used for flow cytometry: CD3e (145-2C11; eBioscience and BioLegend); CD19 (eBio1D3 [eBioscience] and 6D5 [BioLegend]); CD4 (GK1.5), CD5 (53-7.3), CD11c (N418), CD23 (B3B4), CD43 (eBioR2/60), γδ T cell receptor (γδ-TCR) (UC7-13D5), gamma interferon (IFN-γ) (XMG1.2), IgD (11-26c), and IgM (II/41) (all from eBioscience); CD45R/B220 (RA3-6B2), granulocyte-macrophage colony-stimulating factor (GM-CSF) (MP1-22E9), Gr-1 (RB6-8C5), and IL-17A (TC11-18H10.1) (all from BioLegend); and IL-22 (3F11; Genentech). Isotype controls were used to confirm the specificity of staining. For intracellular staining, cells were polyclonally stimulated with 50-ng/ml phorbol myristate acetate (PMA) and 500-ng/ml ionomycin in the presence of brefeldin A (BD GolgiPlug at 1 μg/ml) at 37°C for 5 h, fixed using 4% paraformaldehyde (Thermo Scientific) in phosphate-buffered saline (PBS) for 10 min at 4°C, and then washed in fluorescence-activated cell sorting (FACS) buffer (PBS, 2% fetal calf serum [FCS], 0.09% sodium azide [Sigma-Aldrich]). Cells were permeabilized using PermWash buffer (BD Biosciences) prior to staining. Dead cells were detected by using eFluor506 (eBioscience). For neutrophil quantification via flow cytometry, CountBright absolute counting beads (Invitrogen) were added prior to washing cells and used according to the manufacturer's instructions. Stained cells were analyzed using a FACSAria instrument (BD Biosciences) and FlowJo software (TreeStar).

### P. aeruginosa strains.

The clinical NH57388A strain was provided by N. Hoffmann (University of Copenhagen). This strain possesses a mutation in *mucA* that results in hyperproduction of alginate (14). The mucoid YH5 strain and nonmucoid GRI-1 strain were obtained locally, from a patient with CF and a patient with ventilator-associated pneumonia, respectively. P. aeruginosa strains were maintained in −80°C stocks until required. Prior to use in cell culture, each strain was grown to mid-log phase in Luria-Bertani (LB) broth (Invitrogen) and bacterial concentrations at an optical density at 600 nm (OD_600_) of between 0.4 and 0.6 were quantified by serial dilution and plating to enumerate CFU (GeneQuant Pro spectrophotometer; Amersham Biosciences). Heat-killed P. aeruginosa preparations were produced by heating a known concentration of P. aeruginosa in PBS to 95°C for 10 min.

### Cytokine measurement.

Murine IL-17A, IL-17F, IL-21, IL-22, and IFN-γ were quantified by enzyme-linked immunosorbent assays (ELISAs) (all eBioscience). The lower limits of detection were <4 pg/ml for IL-17A, <15pg/ml for IL-17F, <16 pg/ml for IL-21, <8 pg/ml for IL-22, and <15 pg/ml for IFN-γ. Cytokine levels below the lower limits of detection of the assay were assigned a value of zero.

### Immunochemistry and histology scoring.

Lung sections were stained with brilliant violet 421 anti-B220 antibody (clone RA3-6B2; Biolegend) and anti-CD90.2 antibody (Thy1.2) (clone 53-2.1; Biolegend) conjugated with Alexa Fluor 488-streptavidin (both at a concentration of 1:50). SYTOX green nuclear stain (Invitrogen) was used at a concentration of 1:10,000. The histology scoring system used is shown in Table S2 in the supplemental material.

### Animals.

All mice were used between 12 and 16 weeks of age. IL-17A receptor (IL-17RA) knockout mice (15) were from T. Mitchell, University of Glasgow, and originally supplied by Jay Kolls; μMT mice (16) were from R. Nibbs, University of Glasgow. Each of these lines was on a C57BL/6 background. C57BL/6 mice bred in-house were used as wild-type controls for knockout comparisons. The animal work was carried out under a project license as required by United Kingdom Home Office regulations, as well as scrutiny and approval by an institutional review board.

### Ethics.

The animal studies were approved by the granting of a project license from the United Kingdom Home Office, a ministerial government department that oversees all experimental work with animals in the United Kingdom. The project license number is 60/4361. This work was also reviewed and approved by the University of Glasgow Animal Welfare and Ethical Review Board, under the same license number.

### Mediastinal lymph node, splenocyte, and peritoneal B1a cell stimulation.

Mediastinal lymph nodes and spleens were passed through 80-μm Nitex mesh and red blood cells (RBCs) lysed (RBC lysis buffer; Sigma-Aldrich) to form a single-cell suspension. Cells were either left unstimulated or stimulated with heat-killed P. aeruginosa at a multiplicity of infection (MOI) of 30. Following 3 days of culture, 100 μl of supernatant was removed for cytokine and P. aeruginosa-specific antibody quantification, and cells prepared for flow cytometry.

### Lung homogenate, BAL fluid, and pleural wash samples.

Lung tissue was agitated for 1 h at 37°C with 10 μg/ml DNase (Roche) and 0.65 units/ml Liberase (Roche), passed through 80-μm Nitex mesh, and RBCs lysed to obtain a single-cell suspension. Bronchoalveolar lavage (BAL) fluid and pleural wash samples underwent RBC lysis. Cells were then prepared for flow cytometry.

### P. aeruginosa-specific immunoglobulin quantification.

The ELISA-based method of detecting P. aeruginosa-specific IgM and IgG was adapted from the method of Moser et al. (17). Bound antibody was quantified by detection of biotinylated goat anti-mouse IgM (mu chain specific; Vector Laboratories) or anti-mouse IgG (Fc specific; Sigma-Aldrich).

### Sequence analysis.

Genomes were compared using progressiveMauve.

### Statistics.

Results are presented as median values or, for technical repeats, mean values and standard errors of the means (SEM). Nonparametric statistical tests were used (Mann-Whitney, Kruskal-Wallis, and where appropriate, Dunn's multiple-comparison tests). For parametric testing, Student's 2-sample *t* test was used. Comparisons of animal weight changes were analyzed using repeated-measures ANOVA. Statistical analysis was undertaken using Prism version 6.0 (GraphPad Software). A *P* value of <0.05 was considered significant.

## RESULTS

### Establishment of a model of chronic pulmonary infection with P. aeruginosa.

We adapted the well-established agar bead model of P. aeruginosa infection in mice (18, 19). We used two different clinical strains of P. aeruginosa from patients with CF: YH5, a mucoid isolate, and NH57388A (14), a highly mucoid strain of P. aeruginosa that carries a mutation in the *mucA* gene, resulting in alginate overproduction, a common phenotypic change in P. aeruginosa isolates following chronic infection of the airways in CF patients (20). We performed draft whole-genomic sequencing of the YH5 strain and compared its sequence with the published sequences of the type strain, PAO1 (21), and NH57388A (22). Comparison of the whole genomes (see Fig. S1A in the supplemental material) shows the typical pattern of strain differences in this species, with blocks of highly conserved sequences interspersed with regions of insertions or deletions; note that a segment of the NH57388 genome is inverted relative to the sequence of PAO1. Of the many differences, a notable one is the disruption in NH57388 of the *mucA* gene (see Fig. S1B), which encodes an anti-sigma factor that is responsible for the mucoid phenotype of this strain (14). This gene is retained in PAO1 and YH5. Another significant difference is the loss of some of the genes of the phenazine biosynthesis pathway in YH5 (see Fig. S1C). This pathway is essential for the synthesis of pyocyanin, an important virulence factor for P. aeruginosa (23). These differences are considered further in Discussion.

Following transtracheal delivery of P. aeruginosa-laden beads, virtually all animals recovered completely after anesthesia (4 fatalities within 1 h in 300 procedures). YH5-infected animals remained well for the 14-day period of each experiment, with no differences in clinical score between animals that were given P. aeruginosa-laden beads and those given sterile beads. However, weight loss following bead delivery was significantly greater in mice given the YH5 P. aeruginosa-laden beads than in those receiving sterile beads (24). Following NH57388A inoculation, there were various amounts of early mortality (<4 days), between 0 and 40%. Surviving animals remained well. No animals developed bacteremia following pulmonary infection. At 2 weeks following delivery of beads, NH57388A infection resulted in a mean chronicity rate of 43.4% (standard deviation [SD], 23.9%, and range, 11.1 to 71.5%, for results from 5 experiments with 7 to 10 surviving animals/group). The YH5 strain resulted in a mean chronicity rate of 18.82% (SD, 20.54%, and range, 0 to 37.5%, for results from 6 experiments with 7 to 14 surviving animals/group) at 14 days postinoculation.

As a measure of the inflammatory response following infection, we enumerated neutrophil numbers in BAL fluid samples 2 weeks following infection (see Fig. S2 in the supplemental material) (13). These results showed that animals with viable bacteria within their lungs at 2 weeks following infection had significantly elevated levels of neutrophils within their BAL fluid samples compared to the levels in BAL fluid samples from animals that had received sterile beads or those that had cleared the infection.

The bacterial colony counts in chronically infected animals at 2 weeks after infection were very similar for NH57388A and YH5 (see Fig. S3a in the supplemental material); lung homogenates from animals receiving sterile beads contained no organisms. A notable feature of NH57388A P. aeruginosa colonies recovered from the lungs of chronically infected animals was the appearance of many small-colony variants (see Fig. S3b); this is a common phenotypic variant found in clinical P. aeruginosa isolates and is associated with greater biofilm formation and antibiotic resistance. These small-colony variants were present in over 80% of chronically infected animals and could be seen as early as 4 days after infection. Small-colony variants were not seen following infection with the YH5 strain.

Use of the GRI-1 strain, recovered from a patient with ventilator-associated pneumonia, resulted in marked hemorrhagic pneumonia with death of the infected animals with 24 h, and thus, this strain was not used further in our animal models of chronic infection.

### IL-17RA-dependent signaling mediates resistance to chronic infection with P. aeruginosa.

**FIG 1:**
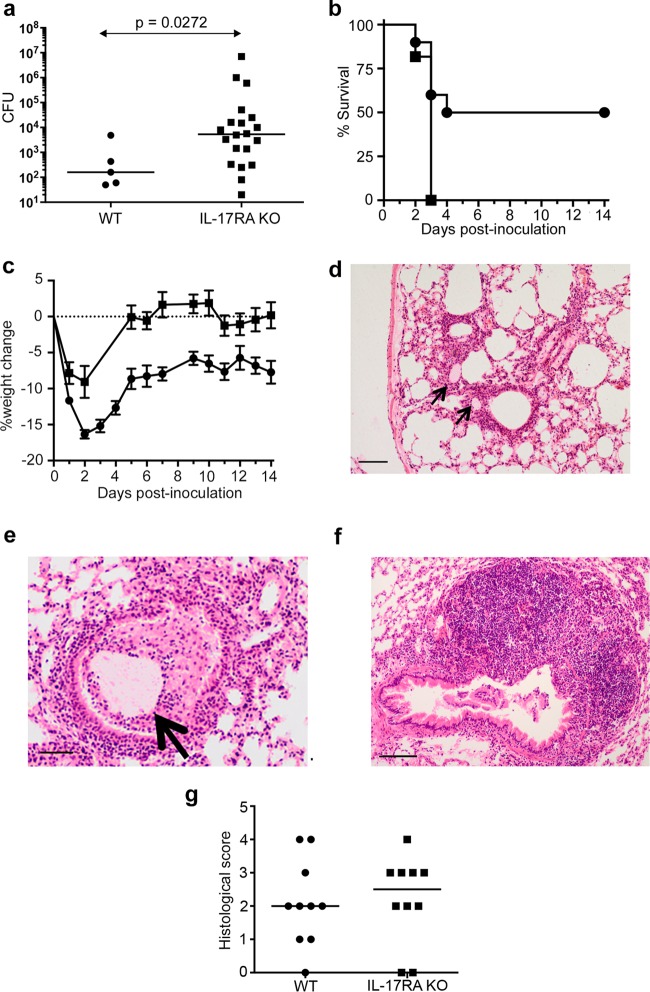
Responses of WT and IL-17RA-knockout animals to pulmonary P. aeruginosa infection. (a) Pulmonary bacterial burdens in animals chronically infected with YH5 at 2 weeks postinfection. Lines indicate median values. *P* value was determined with the Mann-Whitney test from pooled results of two experiments, each with 10 mice per group. (b) Kaplan-Meir survival curves of WT (circles) or IL-17RA knockout (KO) animals (squares) infected with the NH57388A strain (*n* = 10 per group). Significant differences were determined by log rank test; *P* = 0.0092. (c) Weight changes in animals (*n* = 10) remaining chronically infected at 14 days following infection with the YH5 strain; results are for WT (circles) or IL-17RA KO animals (squares). Each point represents the mean weight at that time; error bars show SEM. Differences between the groups are significant at a *P* value of <0.0001 by repeated-measures ANOVA. (d, e) Hematoxylin and eosin (H&E)-stained lung sections from infected WT mice 2 weeks after infection with YH5 strain. Arrows show agar beads. Scale bars show 100 μm (d) or 50 μm (e). (f) Large (>50 cells deep) monocytic accumulations were observed in lungs from IL-17RA KO mice. Scale bar shows 10 μm. (g) Histological scores for indicated animals 2 weeks after infection with YH5 strain. *P* = 0.7266 by Mann-Whitney test.

We compared the responses to P. aeruginosa infection between wild-type (WT) animals and mice lacking the IL-17RA receptor chain that mediates IL-17 family actions (25). Following inoculation with the YH5 P. aeruginosa strain, the infection rates 2 weeks later were 25% in WT animals (5 out of 20 animals) and 100% in IL-17RA knockout mice (20 out of 20 animals), a highly significant difference (*P* < 0.0001, Fisher's exact test). The pulmonary bacterial loads were significantly increased in the IL-17RA knockout animals at 2 weeks following infection (Fig. 1a), but none of the infected animals died or became bacteremic. In striking contrast to the results of infection with the YH5 strain, infection of IL-17RA knockout animals with the NH57388A P. aeruginosa strain resulted in the death of all infected animals within 3 days of infection (Fig. 1b), a statistically significant difference from WT littermates.

All YH5-infected animals, including the subgroup that became chronically colonized, showed an initial drop in weight (Fig. 1c). However, IL-17RA knockout mice showed a smaller initial weight loss and early recovery of their starting weight after infection, a significant difference from the WT animals (Fig. 1c).

### Leukocyte, cytokine, and histological responses following infection.

In wild-type animals, IL-17A and IL-22 were found at low levels in BAL fluid both at 48 h (see Fig. S3c and d in the supplemental material) and 2 weeks (see Fig. S3e and f) following infection, but there was no significant difference between infected animals and sterile-bead-treated controls. Surprisingly, comparison of IL-17RA knockout and WT mice demonstrated that 2 weeks following infection with the YH5 strain, there was no significant difference in absolute neutrophil counts in the BAL fluid (see Fig. S3g); similar results were found following infection with the NH57388A strain (data not shown).

Two weeks after infection, animals showed persistent inflammatory changes in the lung, with localized peribronchial mononuclear infiltrates, and frequently, an agar bead evident in the adjacent airway (Fig. 1d and e). There was no significant difference in overall histological scores between WT and IL-17RA knockout mice (Fig. 1g). However, we did observe areas of very extensive (>50 cells deep) monocytic infiltrates in response to chronic P. aeruginosa infection in IL-17RA knockout animals (Fig. 1f) that were never seen in the WT mice.

### IL-17A production in P. aeruginosa-specific immune responses following infection.

**FIG 2:**
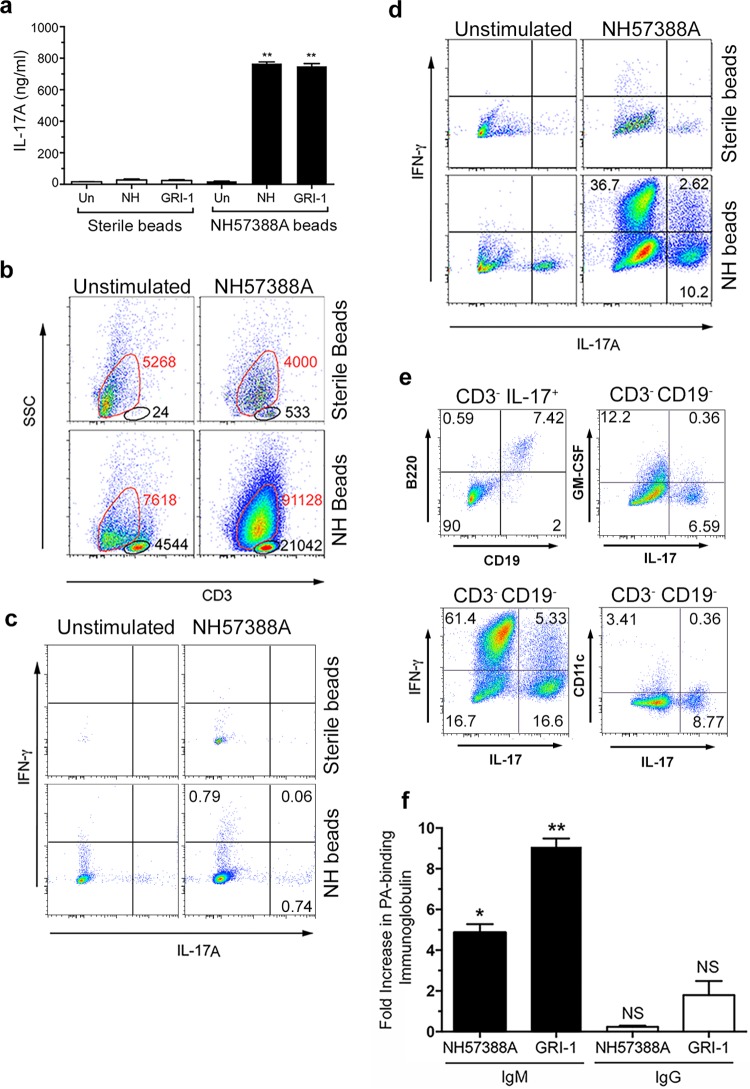
Immune responses following P. aeruginosa infection. Two weeks after infection, mediastinal lymph node cells from WT mice treated as indicated were stimulated *ex vivo* with heat-killed P. aeruginosa strains (MOI of 30) or left unstimulated for 3 days. (a) Levels of IL-17A secretion are shown; bars show mean values and error bars show SEM. **, significantly different from sterile beads at a *P* value of <0.01 by *t* test; Un, unstimulated; NH, NH57388A. Representative results from three separate experiments are shown. (b) Flow cytometry results for stimulated (3 days) mediastinal lymph node cells stained as shown. The CD3^lo-int^ populations are outlined in red, and the CD3^int-hi^ populations are outlined in black. The total number of cells recovered in each gate is shown next to the gate. SSC, side scatter. (c to e) Flow cytometry of lymph node cells as described in the legend to panel B except that cells were gated on the CD3^int-hi^ population (c), the CD3^lo-int^ population (d), or as indicated (e). The percentage of cells in each quadrant is shown. (f) Fold increases in P. aeruginosa-binding IgM and IgG produced by mediastinal lymph node cells from infected animals restimulated with P. aeruginosa strains *ex vivo* compared to the levels produced by unstimulated cells. Columns represent mean values for triplicate wells; error bars show SEM. Columns were compared to a theoretical mean of 1.0 by Student's *t* test. NS, nonsignificant; *, *P* < 0.05; **, *P* < 0.01.

Two weeks following P. aeruginosa infection, there was a marked enlargement of the mediastinal lymph nodes compared to the lymph nodes of control animals (mean values of 3.56 × 10^6^ cells for NH57388A-infected animals versus 2.83 × 10^5^ cells for sterile-bead-treated animals; three separate experiments, *P* < 0.05 by *t* test). *Ex vivo* restimulation of these lymph node cells from infected animals with heat-killed bacteria of the infecting strain, NH73788A, or an unrelated clinical P. aeruginosa strain, GRI-1, for 3 days resulted in robust production of IL-17A, which was not evident in the lymph node cells from animals receiving sterile beads (Fig. 2a). To characterize the cells producing these cytokines, we stained them for a variety of phenotypic surface and intracellular markers. Initially, cells from infected animals were stained for the T cell marker CD3 (Fig. 2b). Comparison with cells stained with an isotype control showed two populations: a very clear CD3^+^ population with low side scatter (Fig. 2b, outlined in black) and a more diffuse population with lower expression of CD3 (Fig. 2b, outlined in red) that overlapped considerably with cells stained with the isotype control. We denoted these populations as CD3 intermediate-high (CD3^int-hi^) and CD3 low-intermediate (CD3^lo-int^), respectively.

Following restimulation of cells from infected animals with NH57388A bacteria, there was a marked expansion of CD3^lo-int^ cells that was not seen in cells from animals that had received sterile beads (Fig. 2b). There was also an increase in the numbers of CD3^int-hi^ cells in the nodes of infected animals; these too increased in numbers in response to restimulation, but to a lesser extent than the CD3^lo-int^ population (Fig. 2b). There was very limited expression of IL-17A or IFN-γ within the CD3^int-hi^ population (Fig. 2c). However, in the CD3^lo-int^ population, there was a significant population of both IL-17A- and IFN-γ-producing cells (Fig. 2d), predominantly from mediastinal lymph node cells of P. aeruginosa-infected animals rechallenged with the infecting P. aeruginosa strain *ex vivo*. The percentage of restimulated cells producing IL-17A that were in the CD3^lo-int^ population was 98% of the combined (CD3^lo-int^ and CD3^int-hi^) population.

We analyzed the IL-17 production within this CD3^lo-int^ population further. Approximately 90% of these cells making IL-17 were also negative for the B cell markers CD19 and B220 (Fig. 2e), consistent with innate lymphoid cells (ILCs) of the group 3 family (ILC3). However, 5 to 10% of this CD3^lo-int^ IL-17^+^ population were reproducibly positive for both of these B cell markers (Fig. 2e). Over 90% of these CD3^lo-int^ CD11c^−^ (a dendritic cell marker) B220^+^ CD19^+^ IL-17^+^ cells were positive for CD5 (see Fig. S4 in the supplemental material), consistent with a B1a population (26). Further characterization of the CD3^lo-int^ CD19^−^ mediastinal lymph node cells expressing IL-17 showed that very few expressed GM-CSF. Over 60% of the CD3^lo-int-^ CD19^−^ cells expressed IFN-γ, but coexpression of IL-17 and IFN-γ was only seen in a minority of these cells (Fig. 2e). The IL-17^+^ CD3^lo-int^ CD19^−^ cells were also essentially negative for the dendritic cell marker CD11c (Fig. 2e) and the NK cell marker NK1.1 (data not shown). Taken together, these data show that the large expansion of cells within the mediastinal lymph nodes 2 weeks following infection with P. aeruginosa was largely composed of cells with the characteristics of ILCs; those expressing IL-17 belong to the group 3 ILC family (27–29). This is considered further in Discussion.

We analyzed P. aeruginosa-specific immunoglobulin production from the mediastinal lymph node cells. We detected five- to ninefold increases in P. aeruginosa-reactive IgM but not IgG in the supernatants from restimulated lymph node cells compared to the levels in supernatants from unstimulated cells (Fig. 2f). This was observed for the *ex vivo* response of lymph node cells to both the infecting strain, NH57883A, and an unrelated strain, GRI-1.

Importantly, we adopted a strict gating strategy to exclude “doublets,” which might otherwise result in the inclusion of T-B cell couples positive for B cell markers, such as B220 and CD19. This removed doublets on the basis of their increased pulse width relative to the side-scatter area (see Fig. S5a to c in the supplemental material) (30). In addition, all analyses were performed under conditions that minimize doublet formation, including the use of cell densities of less than 10^6^ cells/ml, less-than-maximal flow rates, and vortexing of cells prior to the analysis (30). All analyses were performed using the double gating strategy shown in Fig. S5; in practice, this made little difference to the percentages of cells classed as phenotypically B, T, or otherwise. We also evaluated the CD3 staining properties of B220^+^ CD19^+^ cells prior to gating; as shown by the results in Fig. S5d, the B220^+^ CD19^+^ cells (see data in red in Fig. S5d) showed virtually no overlap with the CD3^int-hi^ population (see data in blue in Fig. S5d), supporting the distinction between these populations of cells.

### Parenchymal lung cell responses to chronic P. aeruginosa infection.

**FIG 3:**
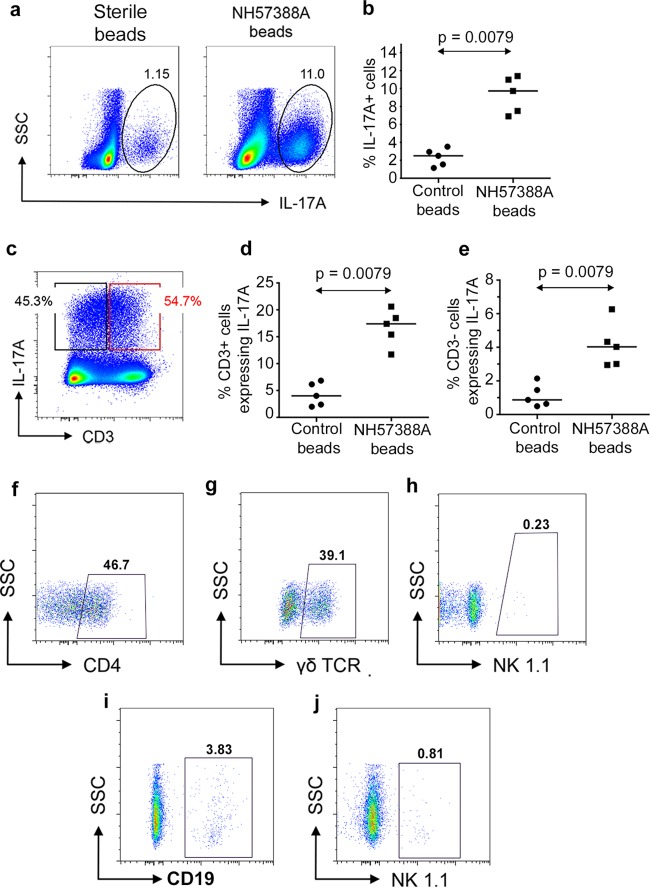
Lung parenchymal responses to P. aeruginosa infection. (a) Representative intracellular expression of IL-17A in lung parenchymal cells 2 weeks following instillation of sterile or P. aeruginosa-laden (strain NH57388A) beads. Numbers show percentages of total cells in ringed areas. (b) Percentages of total lung parenchymal cells expressing IL-17A 2 weeks after introduction of sterile or P. aeruginosa-laden beads. Each symbol represents an individual animal; lines show median values. *P* value was determined by Mann-Whitney test. (c) Representative expression of intracellular IL-17A in CD3^+^ and CD3^−^ populations in lungs 2 weeks following P. aeruginosa infection as assessed by flow cytometry. (d, e) Data are as described in the legend to panel B but show the percentages of CD3^+^ (d) and CD3^−^ (e) cells expressing intracellular IL-17A. (f to h) Flow cytometry results for CD3^+^ IL-17A^+^ cells expressing the indicated markers; boxed areas are deemed positive relative to results for isotype controls. Figures show percentages of the total CD3^+^ IL-17A^+^ population expressing the indicated markers. (i, j) Data are as described for panels F to H but with gating on CD3^−^ IL-17A^+^ cells.

**FIG 4:**
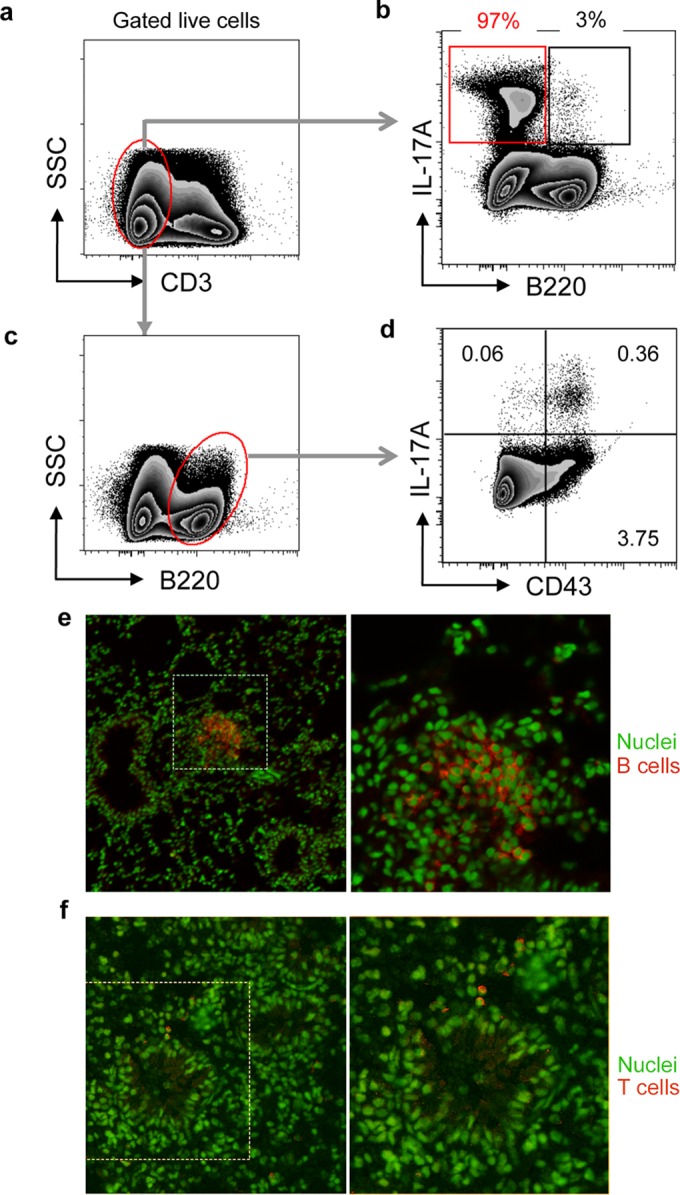
Characteristics of B cells in lungs of animals instilled with P. aeruginosa-laden beads. (a to d) Two weeks after transtracheal instillation of P. aeruginosa-laden agar beads, the lungs were homogenized and cells polyclonally stimulated, extracellularly stained for CD3, B220, and CD43, and then permeabilized and stained for intracellular IL-17A, followed by flow cytometry. (a, b) Representative plots of the expression of B220 and IL-17A by live CD3^−^ cell populations. (b) Values above gates represent percentage of total IL-17A^+^ cells in each gate. (c, d) Representative plots of expression of IL-17A and CD43 by CD3^−^ B220^+^ cells. (d) Values in quadrants represent percentages of total cells. Results shown are representative of two separate experiments. (e, f) Lung sections from animals 2 weeks following infection with NH57388A were immunostained for B cells using B220 staining (e) or for T cells with Thy1.2 staining (f). Immunostaining is indicated in red, and boxed areas are shown enlarged to the right. Nuclei are counterstained green.

Next, we examined the potential of cells within lung tissue to produce IL-17. Lung homogenates showed a significant increase in total cells expressing IL-17 2 weeks following treatment with P. aeruginosa-laden beads in WT mice (Fig. 3a and b), distributed equally between CD3^+^ and CD3^−^ populations (Fig. 3c to e). The CD3^+^ IL-17^+^ population was composed mainly of CD4^+^ (Th17) and γδ T cells in approximately equal numbers; NK/NKT cells were not detected (Fig. 3f to h). The majority of IL-17 expression from the CD3^−^ population is from non-B (B220^−^) cell sources, consistent with an innate lymphoid cell population. B cells constituted 3.83% of the CD3^−^ cells expressing IL-17 (Fig. 3i), with virtually no IL-17 production from NK cells (Fig. 3j). The CD3^−^ B220^+^ population predominantly expressed IL-17 from the CD43^+^ subset, consistent with a B1 cell population (Fig. 4a to d). Lung immunostaining confirmed parenchymal B and T cell responses in P. aeruginosa-infected animals, demonstrating peribronchial mononuclear B cells (B220^+^) and more widely distributed T cells (Thy 1.2^+^) (Fig. 4e and f).

### Response to pulmonary P. aeruginosa infection in B cell-deficient mice.

A small fraction of the IL-17-producing cells in our model of chronic P. aeruginosa infection were B cells, with characteristics of innate B1 cells. Innate B cells have been implicated as important in host defense against bacterial infection, both through their production of natural immunoglobulin that can bind to bacterial pathogens and through differentiating into GM-CSF-producing protective cells (31, 32). We addressed the role of B cells in protection by these mechanisms against chronic P. aeruginosa infection in our murine model. We infected mice that lack cells of the B lineage (μMT mice) using P. aeruginosa-laden beads and compared their responses to those of WT animals.

**FIG 5:**
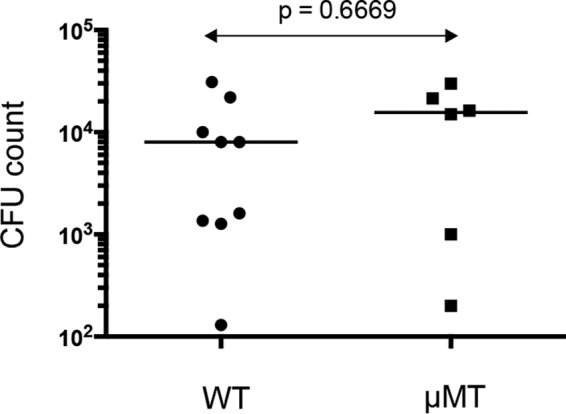
Bacterial counts in WT and μMT mice following infection. Animals were infected with the NH57388A strain. Each symbol represents the result for an individual animal; lines indicate median values. *P* value for comparison between the groups was determined by Mann Whitney test.

There was no difference in mortality between WT and μMT mice 2 weeks following infection (1 in 13 infected μMT animals died, versus 1 in 18 WT animals; *P* = 1.0 by Fisher's exact test). The proportions of animals chronically infected 2 weeks after infection were also not significantly different between the groups (6 of 12 μMT animals versus 9 of 17 WT animals; *P* = 1.0 by Fisher's exact test). Furthermore, the pulmonary bacterial burdens in chronically infected animals were not significantly different between the groups (Fig. 5). There was no difference between WT and μMT mice for IL-17 production from P. aeruginosa-stimulated mediastinal lymph node cells at 14 days after infection (data not shown).

## DISCUSSION

Here, we have shown that IL-17 family cytokines play a crucial role in preventing chronic infection of the airways with P. aeruginosa in a murine model of infection. The change from intermittent to chronic infection of the airways with P. aeruginosa is a key transition point in patients with CF; delay of this stage would likely prolong life expectancy in patients with CF. Thus, enhancing IL-17 actions at this point in the clinical course of CF could be a potential therapeutic target.

IL-17 family members have a potent effect on neutrophil production and mobilization (15, 33). Although we found small reductions in blood neutrophil counts between WT and IL-17RA knockout animals before and after infection (data not shown), there was no difference in BAL fluid neutrophil counts between these groups of animals following infection. However, pulmonary neutrophil recruitment may still play a critical role in preventing chronic infection via a very early IL-17-dependent neutrophil influx. In addition, direct IL-17 activation of neutrophils was recently shown to be required for optimal fungal killing (34). The protective effect of IL-17 in the pulmonary P. aeruginosa infection model could also result from the ability of IL-17 to induce antimicrobial peptides, such as defensins and the S100 proteins; indeed, in ocular infections, human β-defensin 2 plays an important role in host defense against P. aeruginosa (35). In addition, IL-17 induces proinflammatory cytokines like IL-6 and tumor necrosis factor alpha (TNF-α). The lack of weight loss seen in infected IL-17RA knockout mice may reflect the lack of such inflammatory cytokine production, since these cytokines are associated with loss of body mass (36).

We identified a number of different cellular sources that produce IL-17 in this model of infection. In the draining mediastinal lymph nodes following infection, we found a considerable expansion of cells that predominantly had the characteristics of group 3 ILCs. Specifically, given their production of IL-17, these are likely to be lymphoid tissue inducer (LTi) cells. Further characterization using the ILC3 markers CD127 and RORγt will be required to establish the identity of these cells beyond doubt. ILC3/LTi cells have been implicated in host resistance to extracellular bacteria, chronic inflammation, and tissue repair. Recently, group 3 ILCs have been shown to present antigen and to contribute to the control of CD4^+^ T cell responses to commensal bacteria (37, 38). The expansion of group 3 ILCs in the mediastinal lymph nodes has been described previously in a helminth infection model (39). This study found that in mesenteric lymph nodes, the group 3 ILCs migrated in a CCR7-dependent fashion from the intestine. The origin of the group 3 ILCs that migrate to mediastinal lymph nodes is not clear, especially as the lung has a very low number of these cells. The function of these cells in this location is also not clear. Neutrophils are recruited to regional lymph nodes in infection and inflammation. In this location, they potentially fulfill a number of roles, including limiting pathogen escape, modulating subcapsular sinus macrophage numbers, and influencing dendritic cell maturation and antigen presentation (reviewed in reference 40). IL-17 family cytokines released by group 3 ILCs within draining lymph nodes would thus be one mechanism whereby neutrophils could be recruited to this site during an infection. Further work will be required to explore these possibilities.

We found a small but significant population of B cells that produced IL-17 in the mesenteric lymph nodes following infection. B cell production of IL-17 has been found in a model of Trypanosoma cruzi infection in mice (41). Our study suggests that B1 cells are a source of this cytokine. B1 cells are the predominant B cell in pleural and peritoneal compartments and continuously traffic into these areas by a CXCL13-dependent pathway (42). Following activation, they migrate to regional lymph nodes and intestinal lamina propria (26, 43). The accumulation of IL-17-producing B1 cells in mediastinal lymph nodes following infection reported here may reflect this activation-induced cell trafficking. Immature plasma cells (plasmablasts) may also express CCD19, B220, and CD43 and, thus, may also be the B cells producing IL-17 within the mediastinal lymph nodes described here (44). Further work with genetic marking of distinct lineages will be required to identify these cells unequivocally. These mediastinal node cells from P. aeruginosa-infected animals require further stimulation with P. aeruginosa to expand the population of B1a cells and to produce secretion of IL-17. This migration and subsequent division in response to P. aeruginosa is likely produced via Toll-like receptor (TLR) stimulation with lipopolysaccharide (LPS) from the Gram-negative organisms, as has been shown previously (45). However, given that mice lacking B cells showed no defect in the incidence of chronic infection with P. aeruginosa, these B1 cells are dispensable as a source of protective IL-17.

Within lung parenchyma following infection, IL-17 was produced from CD4^+^ cells with properties of Th17 cells, as well as γδ T cells. Both these cell types were increased in the lung following chronic infection. The generation of P. aeruginosa-specific Th17 cells by a pseudomonal vaccine may thus be of importance in providing protection against chronic infection.

The P. aeruginosa strains used here were both derived from CF patients but showed important differences in the model (Fig. 1). Apart from the known difference in alginate production in the NH57388A strain, genome sequencing revealed multiple genetic differences between this strain and YH5 (see Fig. S1 in the supplemental material). The alginate overproduction in NH57388A is due to a deletion of part of the *mucA* gene, which encodes a MucA anti-sigma factor (22). Also of interest is that YH5 has lost the genetic machinery necessary to synthesize pyocyanin. This could account for its relative lack of virulence compared to that of the NH57388A strain in the IL-17RA knockout animals, but further work will be required to determine which of these genetic differences are most important.

Chronic P. aeruginosa infection is a significant problem not only in CF but also in COPD (46) and non-CF bronchiectasis (47). Delaying the onset of chronic infection in such patient groups would represent a very valuable therapeutic goal. Antibiotics can delay this progression but cannot prevent this transition. A number of vaccines against P. aeruginosa have been developed to attempt to prevent chronic infection; as yet, none of these have shown clinical efficacy (48). The work described here opens a new perspective on the prevention and treatment of such P. aeruginosa infection. Potential therapies based on augmenting IL-17 action—through vaccination or otherwise—could be of great value in delaying or preventing chronic P. aeruginosa infection in chronic lung disease.

## Supplementary Material

Supplemental material
