# Supplementary material for: Interleukin-17 Is Required for Control of Chronic Lung Infection Caused by Pseudomonas aeruginosa
Source: Infect Immun. 2016 Nov 18;84(12):3507–16. doi: 10.1128/IAI.00717-16 (PMC5116727; doi:10.1128/IAI.00717-16)
Supplement: Supplemental material [file supp_84_12_3507__index.html]

Interleukin-17 Is Required for Control of Chronic Lung Infection Caused by Pseudomonas aeruginosa — Supplemental material 

# Interleukin-17 Is Required for Control of Chronic Lung Infection Caused by Pseudomonas aeruginosa

## Supplemental material

- Supplemental file 1 -

  Fig. S1. Genomic comparisons of PAO1, NH57388A, and YH5. Fig. S2. Bronchoalveolar neutrophils in response to sterile and *Pseudomonas aeruginosa* laden beads. Fig. S3. Responses of animals following infection. Fig. S4. CD5 staining of B cell population within mediastinal lymph nodes. Fig. S5. Gating strategy for selecting singlets and CD3+ and CD3− cells. Table S1. Severity scoring system for infected animals. Table S2. Histological scoring system for inflammation in lungs of mice treated with agar beads.

  PDF, 3.1M
