# Supplementary material for: Interleukin-17 Is Required for Control of Chronic Lung Infection Caused by Pseudomonas aeruginosa
Source: Infect Immun. 2016 Nov 18;84(12):3507–16. doi: 10.1128/IAI.00717-16 (PMC5116727; doi:10.1128/IAI.00717-16)
Supplement: Supplemental material [file IAI.00717-16_zii999091898so1.pdf]

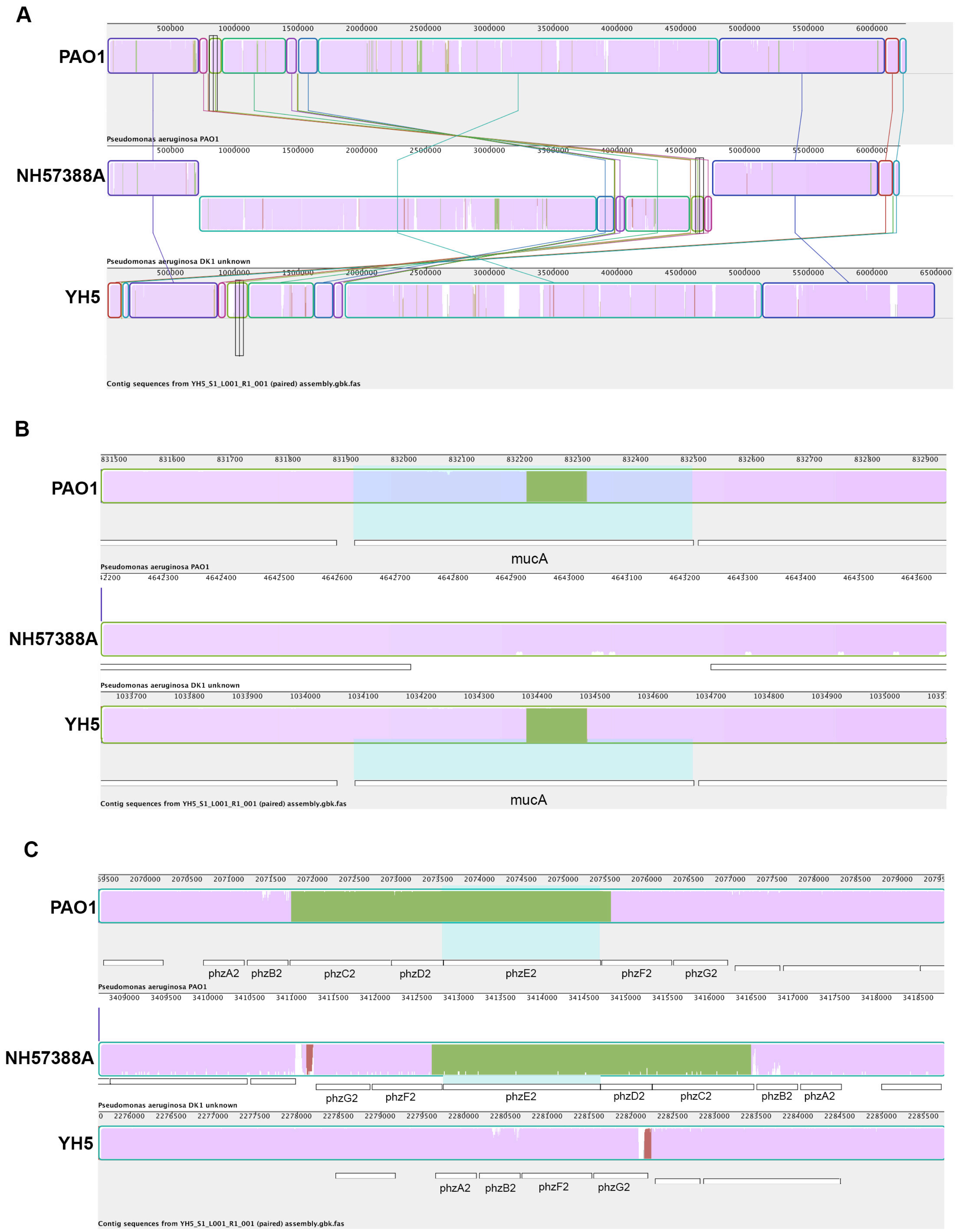

Figure S1. Genomic Comparisons of PAO1, NH57388A and YH5. (A), whole genomes compared using Progressive Mauve. Areas of identity are shown in solid lilac with corresponding blocks joined by vertical lines. Note a large segment of the NH57388A genome is inverted relative to the type strain PAO1. (B) comparison of the genomic region containing the *mucA* gene. Solid green indicates a region common to PAO1 and YH5 that is missing in NH57388A. (C) comparison of the phenothiazine gene operon. The solid green region is common to both PAO1 and NH57388A (inverted in this area) but missing in YH5.

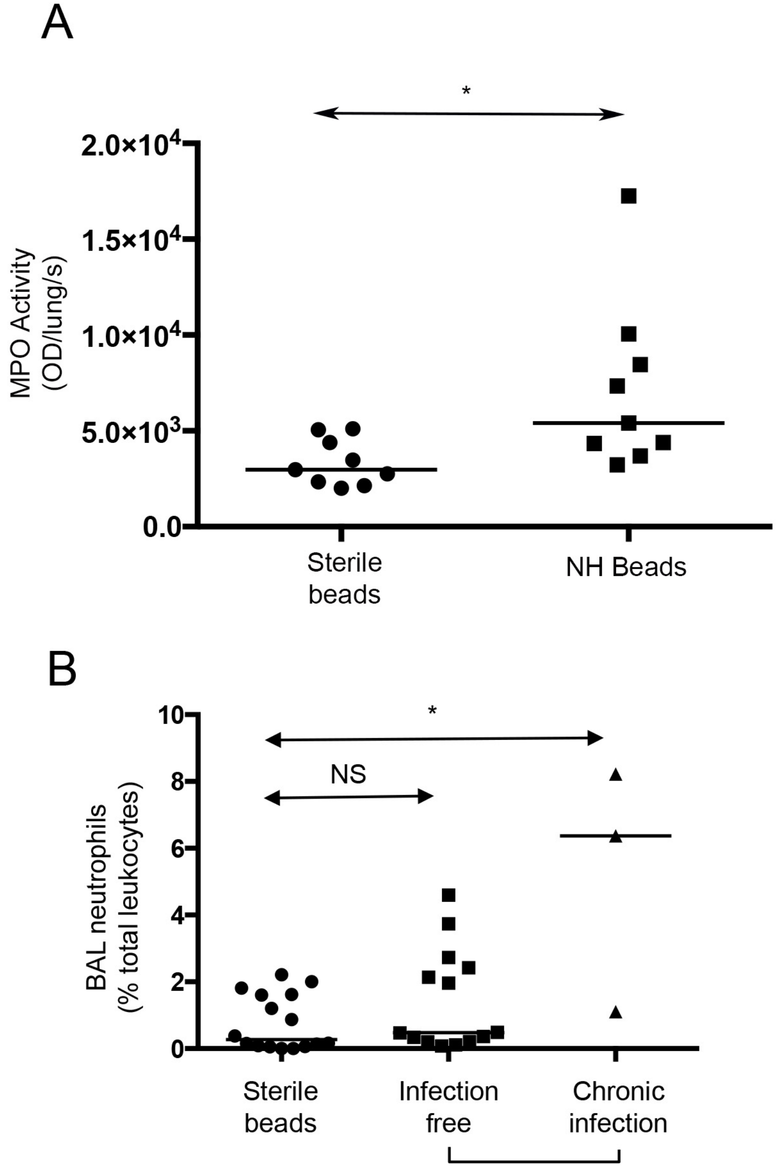

Figure S2. Bronchoalveolar neutrophils in response to sterile and *Pseudomonas aeruginosa* laden beads

BAL neutrophils (MPO activity (A) or percent of total leukocytes (B)) in animals treated with sterile beads were compared with mice that had received NH57388A laden beads (A) or had cleared PA (infection free) or remain infected (chronic infection) at 2-weeks following treatment with Yorkhill 5-laden (B) beads. Combined results of two separate experiments. Line indicates median. Differences between groups were evaluated by a Kruskal-Wallis (KW) test with pairwise assessments of differences between sterile-bead and PA-laden bead treated groups made using Dunn's multiple comparison test. NS, not significant. *, significance difference < 0.05.

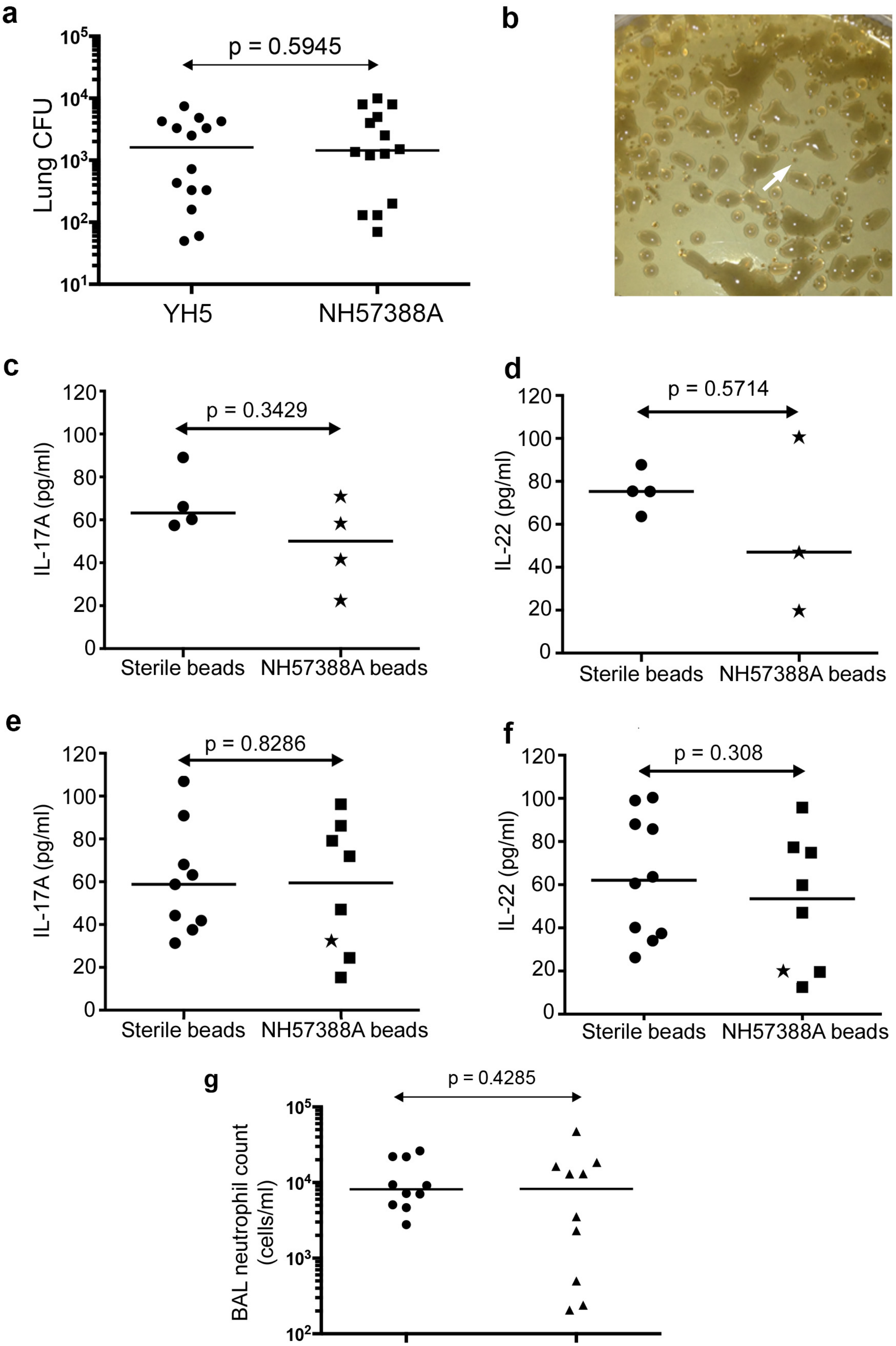

Figure S3. Responses of Animals following Infection (A) Bacterial counts recovered from the lungs of animals 2 weeks following infection. Each symbol is an individual animal; p value is for mann Whitney test between the groups. (B). Representative view of colonies recovered from lungs of mice infected with the NH57388A strain. Arrow shows small colony variant.(C) – (F), Levels of IL-17A (C, E) and IL-22 (D, F) in BAL from sterile or PA-laden bead treated animals after 48h (C, D) and 2 weeks (E, F). Each point represents an individual animal; line indicates median. * indicates animal with ongoing pulmonary PA infection. P-values shown are comparisons by Mann-Whitney test. (G) BAL neutrophil counts 2 weeks after infection in the indicated mice strains. Each point represents an individual animal; line indicates median. P-values shown are comparisons by Mann-Whitney test.

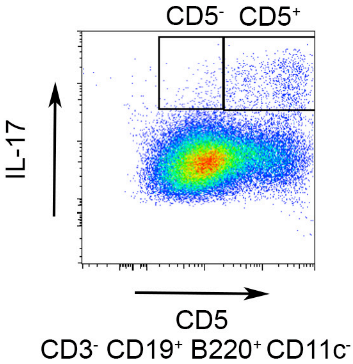

Figure S4. CD5 staining of B cell population within mediastinal lymph nodes. Lymph node cells from infected animals were stained for surface markers and intracellular IL-17 and gated as shown using flow cytometry. The $CD5^-$ and $CD5^+$ populations are shown. The $CD5^+$ population was typically ~90% of the IL-17^+ cells. Representative of two separate experiments.

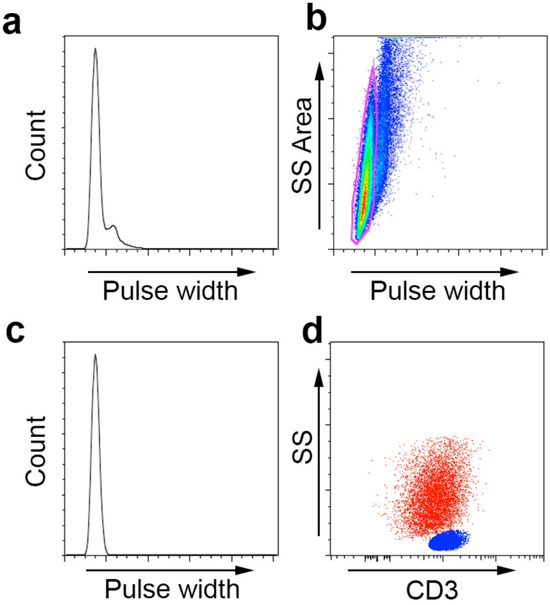

Figure S5. Gating strategy for selecting singlets and CD3⁺ and CD3⁻ cells. Lymph node cells from infected animals were stained for the markers shown in Fig 2 c-f and analyzed for pulse width and count (**a**), and pulse width and side scatter area (SS Area) (**b**). Cells with pulse width characteristics of singlets were gated as shown by the pink gate. (**c**) cells within the gate shown in **b** re-analysed for pulse width and count. (**d**) CD3 staining characteristics of lymph node cells that are B220⁺ and CD19⁺ (shown red) compared to cells that are CD3^{int-hi} (shown blue).

**Condition**	**Level**	**Description**	**Score**
Normal		Normal stance and movement	0
Hunched	1	Slightly hunched stance	1
	2	Pronounced hunched stance	2
Starey Coat	1	Mild piloerection of coat, mainly around back of neck	3
	2	Marked piloerection over whole body	4
Lethargic	1	Slightly slower movement than usual	5
	2	Obviously slower movement	6
Moribund		Unwillingness to move when encouraged to do so	7
Weight loss		> 20% initial body weight	7

Table S1. Severity scoring system for infected animals.

Animals with a total score of ≥ 7 were culled.

Score	Peribronchial infiltrate	Alveolar involvement
0	**None**	**None**
1	**Mild** (infiltrate ≤ 4 cells thick)	**Mild** (patchy increased cellularity/thickening)
2	**Moderate** (infiltrate 5-10 cells thick)	**Moderate** (25-50% visualized lung with increased cellularity/thickening)
3	**Severe** (25-50% visualized lumens)	**Severe** (>50% visualized lung with increased cellularity/thickening)
4	**Diffuse** (>50% visualized lumens)	

Table S2: Histological scoring system for inflammation in lungs of mice treated with agar beads

Score applied to whole lung section, scored at x10 magnification.
